# Daily School Physical Activity Is Associated with Higher Level of Physical Activity Independently of Other Socioecological Factors

**DOI:** 10.3390/sports8080105

**Published:** 2020-07-29

**Authors:** Amanda Lahti, Björn Rosengren, Jan-Åke Nilsson, Magnus Dencker, Magnus Karlsson

**Affiliations:** 1 Clinical and Molecular Osteoporosis Research Unit, Departments of Clinical Sciences and Orthopedics, Skåne University Hospital, Lund University, SE-20502 Malmo, Sweden; bjorn.rosengren@med.lu.se (B.R.); jan-ake.nilsson@med.lu.se (J.-Å.N.); magnus.karlsson@med.lu.se (M.K.); 2 Departments of Clinical Physiology, Clinical Sciences, Skåne University Hospital, Lund University, SE-20502 Malmo, Sweden; magnus.dencker@skane.se

**Keywords:** school, relative-age effect, public health, intervention

## Abstract

Only one fifth of children aged 11–17 years are physically active for 60 min (min)/day. As physical activity (PA) levels track from childhood to adulthood, it is important to establish healthy PA behavior early in life. This study aims to evaluate whether daily school PA is associated with objectively measured PA independently of other socioecological factors. This study includes 209 children (120 boys) aged 9.8 ± 0.6 (mean ± SD) years from four government-funded schools in Sweden. One school including 113 children (70 boys) had 40 min of daily school PA (intervention) and three schools including 96 children (50 boys) had 60 min of school PA/week (control). PA was measured during four serial days with accelerometers. General PA (GPA) was defined as mean counts per minute (cpm). Socioecological factors were collected by questionnaires, and anthropometric traits by measurements. Analysis of covariance (ANCOVA) was used to test whether sex, age, relative age, body height, fat mass, lean mass, screen time activity, parental educational level, parental attitude towards PA, parental PA, sibling(s)’ PA, living in a house or apartment, and whether the child was allocated to 40 min daily school PA or 60 min school PA/week, was independently associated with GPA. Daily GPA was found to be 686.9 ± 211.9 cpm. Independently of the other included factors, daily school PA was associated with +81.8 (15.7, 147.8) cpm compared with 60 min PA/week. This study infers that daily school PA is an appropriate strategy to promote PA in 10-year-old children, independently of different socioecological factors.

## 1. Introduction

More than a quarter of the global adult population is insufficiently physically active, and only one fifth of children aged 11–17 years meet the World Health Organisation (WHO)’s recommendation of 60 min (min) of physical activity (PA)/day [1]. Benefits of PA in children include increased muscular strength and respiratory fitness, lower body fat percentage, and better cardiovascular, metabolic, and bone health [2]. Promotion of childhood PA is of importance as PA levels track from childhood to adulthood [3,4], and adequate childhood PA levels may protect against obesity and other chronic diseases later in life [5].

Schools may be a beneficial arena to promote PA in children as it is possible to reach all children in society, regardless of socioeconomic settings. Previous studies conducted in a Swedish middle-class area, which included four government-funded schools that followed children annually during the compulsory school years, found that daily school PA is associated with higher PA levels during [6], as well as three years beyond the compulsory school years [7]. Conversely, over the past century, Swedish schools have reduced time devoted to physical education in favor of academic subjects [8]. This trend is also found globally, as one study in the United States found that student participation in physical education classes declined from 3.6 days to 3.0 days/week during 1984 and 1994 [9].

Socio-ecological models were developed during the 1950s to understand, as well as to guide interventions aiming to change behavior on a population-based level. Between 1965 to 2005, the combination of efforts at all levels of the socio-ecological model contributed to a drop in smoking rates among adults in the United States of 21 percent [10]. The model was also adopted later on to improve other health behaviors, including childhood PA [10].

According to the socio-ecological model, there are several factors that may be determinants of PA, such as individual (e.g., sex), behavioral (e.g., sedentary time), social (e.g., family), and environmental (e.g., availability of PA facilities) factors [11]. As many factors influence PA behavior in children, it is hypothetically possible to target several other factors apart from school PA in order to promote childhood PA. In addition to scheduled school PA programs, family programs [12], recess programs [13], and active commuting programs [14] have been evaluated, but none of the cited studies compare the influence of different possible explanatory factors on increasing PA levels in school-aged children. A recent review article further justifies this study when reporting that only 47–65% of different intervention trials have been reported effective on children’s PA levels, and that school-based PA interventions may possibly increase the level of PA in children [15]. In other words, should interventions aiming to increase childhood PA target school PA or focus on other factors?

This study aims to evaluate whether there is any independent association between (i) 40 min daily school PA (compared with 60 min school PA/week) and (ii) a variety of other socioecological factor (s), and objectively measured general physical activity (GPA).

## 2. Material and Method

The POP study is a population-based prospective controlled intervention study that followed children during the compulsory school years and onwards, to examine whether the daily scheduled 40 min of school PA induced short- and/or long-term health benefits [7,16,17]. This study utilized data from the POP study 2 years after baseline when the children were mean 10 (range 8–11) years old.

### 2.1. Study Participants

**Figure 1:**
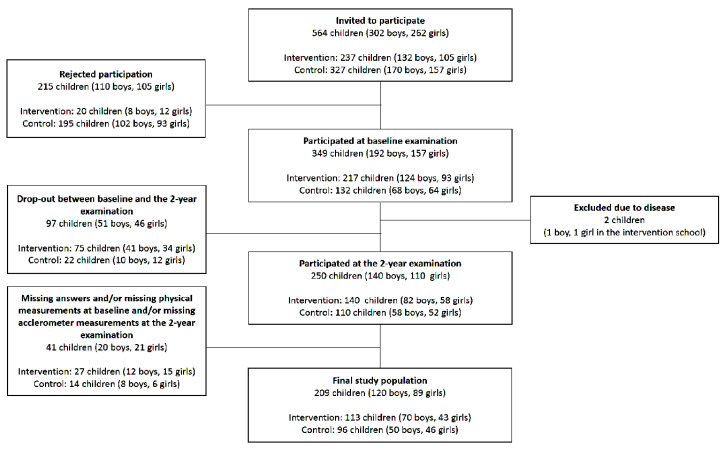
Flow-chart of the study population. To be included in this study, the participants had to have valid data from both the baseline and the 2-year follow-up exam.

In the POP study, all 302 boys and 262 girls in first- or second-grade in four government-funded neighboring schools within a defined geographical urban middle-class area were invited to participate. The children were allocated to each school depending on their residential address and did not actively choose to attend school at the start of the school year. At baseline, 192 of the boys (64%) and 157 of the girls (60%) (age 7.8 ± 0.6; mean ± SD) accepted participation. Two children (one boy and one girl in the intervention school) were excluded due to diseases affecting their physical ability. At the 2-year follow-up examination, 140 boys and 110 girls participated. Twenty boys and 21 girls were excluded due to missing accelerometer data, and incomplete physical measurements and/or answers in the questionnaire. This study thus included 120 boys and 89 girls (Figure 1) from the original POP study cohort.

### 2.2. The Intervention

**The first school we asked agreed to be an intervention school. The three others were then allocated as control schools. That is, there was no randomization before the start of the study. The intervention** school increased the duration of school PA to 40 min/school day, whereas the three remaining schools continued with the Swedish standard of 60 min/week in one or two sessions. The duration of 40 min/school day was chosen as it was practically possible to include in the ordinary schedule. The intervention included a variety of activities within the school curriculum, such as running and ballgames. The sessions were led by the ordinary teachers.

### 2.3. Measurements and Procedures

PA was measured with the Actigraph accelerometer (ActiGraph, Pensacola, FL, USA, model 7164) that measures vertical movement in counts per minute (cpm) during 10-second periods. The equipment was not water-resistant. The children were instructed to wear the accelerometer on the right hip over four consecutive days. Measurements were considered acceptable if the accelerometers had been worn for a minimum of three separate days with a minimum of eight hours of valid recording/day. The accelerometer measurements took place during the autumn school term (August–December) in close relation to the annual evaluations of the POP study. Of the 209 included children, 169 children (81%) fulfilled four days, and 40 children (19%) three days of minimum eight hours/day of recording. Detailed description of the accelerometer and data collection procedure have been described in a previous study [18]. Based on previous studies [18,19,20], we chose to estimate GPA as mean counts per valid minute of recording.

Socioecological factors that have previously been found to be associated to PA in children [11,21,22,23] and were available in the POP study was included in the study. According to a socioecological approach, the factors were classified into three domains: biological, social, or environmental [10]. Age, relative age (i.e., born during January–June or July–December), and sex was calculated from the children’s personal code number. Body height (cm) and body weight (kg) were measured by using standard equipment (Harpenden Stadiometer, Holtain Ltd, Pembrokeshire, UK) and an HL 120 electric Scale (Avery Berkel, West Midlands, UK). Body mass index (BMI) was calculated as weight/height^2^ (kg/m^2^). Proportions of total body fat and total body lean mass (%) were measured by dual energy X-ray absorptiometry (DXA) Lunar DPX-L version 1.3z (Biodex Medical Systems Inc, New York, NY, USA). Together with a parent or member of the research staff, the children answered questions on social and environmental factors; whether they enjoyed/disliked physical education classes (yes/no) and screen time activity (h/day). The parents reported their highest level of education, their duration of organized leisure-time PA, whether the family lived in a house or apartment, and whether the child had any sibling(s). If the child had sibling(s), we also asked whether the sibling(s) was or were active in a sport association. The parents also answered whether they agreed/partly agreed/disagreed with the statement, “In our family it is important to exercise”.

The answer to the question on screen-time activity was converted from h/day to h/week by multiplication by seven. Median screen-time activity was 14 h/week, and this variable was dichotomized into high (>14 h/week) or low (<14 h/week) and excluded missing values (*n* = 15). Parental attitude (“In our family it is important to exercise”) was also dichotomized into agree/partly agree or disagree with one, and excluded missing values (*n* = 15). The question, “Do you think physical education is fun?” was excluded, as 194/209 children (93%) answered “yes” (11 children (5%) did not answer the question and four children (2%) answered “no”), and the question was thus not discriminative.

In summary, the final model was adjusted for sex, age, relative age (i.e., born on first or second half of the year), body height, fat mass, lean mass, duration of children’s screen time activity, parental educational level, parental attitude towards PA, mean duration of parental PA, having a physically active sibling or not, living in a house or apartment, and whether the child attended the school with daily PA or with 60 min PA/week. All factors are presented in detail in Appendix A.

Previous drop-out analysis at baseline [18] that utilized the general school health data register found similar body weight, body height, and BMI in children who at baseline both agreed and disagreed to participate in the study.

### 2.4. Data Analysis

IBM SPSS (Version 23, Chicago, IL, USA) was used for statistical analyses. Descriptive data are presented as numbers (*n*), proportions (%), or means ± standard deviations (SD) and inferential statistics as means with 95% confidence intervals (95% CI). Analysis of covariance (ANCOVA) was used to determine associations between objectively measured PA and each specific factor both unadjusted and adjusted for all other factors included the model. Statistical significant difference was defined as *p* < 0.05. Written consent from guardians and children was obtained before the start of the study. The study was conducted according to the Declaration of Helsinki, approved by the Ethics Committee of Lund University, Sweden (LU 453-98; 1998-09-15), and registered as a clinical trial (ClinicalTrials.gov.NCT00633828).

## 3. Results

Descriptive characteristics of the included children are presented in Table 1. Daily GPA was 686.9 ± 211.9 cpm (mean ± SD) (boys 738.2 ± 230.8, girls 617.7 ± 160.2). The included factors explained 28% of the variance in GPA. The daily school PA intervention was associated with +81.8 (95% CI 15.7, 147.8) cpm compared to children having PA 60 min/week (control schools). That is, 200 min of weekly school PA was, independently of the other included factors, associated with more general PA, as estimated through objective accelerometer measurements, than having 60 min weekly school PA. In addition, the female sex was associated with 66.1 (9.3, 122.9) less cpm compared to the male sex; each had a 10 cm shorter body height with 69.0 (24.6, 113.3) less cpm; and each year of older age was associated with 54.8 (1.4, 108.2) less cpm (Table 2). 

## 4. Discussion

This study found that 40 min daily school PA, independently of several other socioecological factors, is associated with more objectively measured PA compared with having 60 min school PA/week. This emphasizes that school is a feasible arena to promote PA in 10-year-old children. The secondary findings suggest that children of female sex, older age, and shorter body height may need extra support to be physically active.

Previous POP studies have found several health benefits of daily school PA, such as favorable bone [16] and cardiovascular health [24], improved academic results [25], and higher level of PA, both during [6] and beyond [7] the program. However, the cited POP studies are often limited due to confounding factors and criticized for being conducted in a socioeconomically privileged area, which may bias the results. The question of whether interventions aiming to increase PA in children should target schools rather than other potential explanatory factors [11,22,23] therefore remains uncertain.

This study provides new knowledge, as the independent association between daily school PA and higher GPA persisted after also adjusting for a variety of potentially explanatory factors. This study infers that it is the school PA per se, and no other socioecological potentially explanatory factor(s), that promotes GPA in children. Our results support the conclusions in a review article that school-based PA interventions have the potential to increase PA in children [15]. The reason why schools can be a feasible arena to promote PA in children could possibly be due to how children spend a large proportion of their waking hours in school, and how schools reach all children in society, including those that do not already have an interest in sport or have parents who encourage leisure-time PA [26]. Taken together, daily school PA throughout compulsory schooling is probably a proper strategy to promote PA in 10-year-old children, independently of different socioecological factors.

A recent report involving almost a thousand of Swedish 15-year-olds showed that less than one third of children with parents without post-secondary degrees and with low income participated in organized leisure-time PA, compared to four fifths of children with parents without post-secondary degrees and high income [27]. The cited study shows a socio-economic difference in participation rate in organized leisure-time PA in advantage to children of Swedish origin, living in wealthy families, and having well-educated parents, and that expensive leisure time PA excluded children without these socioeconomic advantages. In this study, school PA was independent from several other socioecological factors associated with more GPA, and this result led to the speculation of daily school PA also being a proper strategy to increase PA in children living in less privileged areas. Future studies should examine the generalizability of the results.

PA habits are known to track from childhood to adulthood, and start to develop early in life [3,4]. One study found that the inter-age correlations and stability coefficients from age 9 to 12 years are lower than those from age 18 to 21 years, indicating that PA is less stable in childhood and early adolescence than that in adulthood [4], and that healthy PA habits should therefore be established as early in life as possible. The reason for the tracking phenomenon may be that in adulthood, people continue with activities they engaged in at a young age automatically, without awareness of the behavior [28]. Another possible explanation is that childhood PA benefits basic motor skills that makes it easy to maintain PA habits and/or start again after a possible break [28]. The tracking phenomenon has been observed in the POP study, where children at the intervention school remained more physically active than controls also beyond termination of compulsory school [7]. Taken together, it is likely that daily school PA leads to more PA later in life. This is important, as an active lifestyle can prevent many diseases, such as type 2 diabetes, some cancers, and coronary heart disease later in life [1], and probably also provide relief to the substantial economic burden caused by inactivity-related diseases [29].

Beyond school PA, female sex, older age, and shorter body height were factors associated with lower level of GPA. It is often suggested that PA levels during childhood decline with age and more so in girls than boys, resulting in an apparent sex difference from puberty and onwards [30]. This study supports this notion, as both female sex and older age were associated with lower GPA. The sex discrepancy also remained after adjustment for common biological differences between boys and girls (i.e., lean mass, fat mass, and body height) [31], which may highlight the importance of sociocultural factors rather than biological attributes for this divergence between boys and girls.

The relative age effect refers to a bias in youth elite sport in favor of children born early in the year due to advantages in growth and maturation compared to those born later in the year [31,32]. As shorter body height (probably as an expression of less advanced growth and maturation [31]) is associated with lower GPA, a relative age effect may also be present for PA in the general population of children. A previous study found no association between body height and duration of PA in 8-year-old children [33], and a speculation is therefore that this phenomenon may not appear until around puberty, when differences in maturation result in greater individual variance and when PA becomes more competitive.

Opponents to daily school PA have also raised the question of whether it is fair to force children who dislike PA to partake in mandatory school PA during all compulsory school years. It is therefore important to highlight that 93% of the children in this study answered that they enjoyed school PA.

During recent decades, much attention has been given to the adverse health effects of sedentary activity. Incremental evidence suggests that sedentary activity and PA do not necessarily displace one another [34,35,36]. The current study indirectly supports this view, as we were unable to find an independent association between sedentary activity (defined as screen time) and PA. Interventions that aim to decrease sedentary activities therefore need not necessarily target the same factors as interventions that aim to increase PA.

Study strengths include the use of accelerometers to estimate PA, the population-based study design, and the inclusion of a variety of factors within several domains of the socioecological model that explain as much as 28% of the variance in PA. It is also a strength that we use a comprehensive framework and pinpoint the most important factors over a range of different socioecological factors that in previous studies have been reported to be associated with PA. Study limitations include the homogeneity of the population regarding age and socioeconomic background. It would have been advantageous to include more factors of interest, such as self-efficacy, season variability, and transportation to school [11,22,23]. There is a risk of bias, as children may have tried to be more physically active than otherwise during the accelerometer measurements, and only the most motivated children may have participated in the study. Unfortunately, no accelerometer measurements were conducted at baseline, making it impossible to evaluate a possible change in PA levels over time.

Even if accelerometers measure objective GPA, we may still have underestimated the true PA, as the equipment only measured vertical acceleration and was not water-resistant, thus missing such activities as cycling and swimming. It would also have been advantageous to register accelerometer data for a longer duration than four days [37]. We must also acknowledge that this is a cross-sectional hypothesis-generating study where causality cannot be established.

In summary, this study supports that daily school PA is a proper strategy to increase PA in 10-year-old children, with results that support the inclusion of more PA during the compulsory school years. In addition, we also found that children of female sex, older age, and shorter body height may need extra support to be physically active. Future studies should verify our findings in other socioeconomic areas and evaluate whether interventions in girls, older children, and children with shorter body height could improve the level of PA.

## Figures and Tables

**Table 1:** Descriptive data on all included children mean 2 years after baseline. Data are presented as mean ± standard deviations or numbers with proportions (%) within brackets.

	40 min Daily School PA (Intervention)		60 min School PA/Week (Control)	
	Boys (*n* = 70)	Girls (*n* = 43)	Boys (*n* = 50)	Girls (*n* = 46)
**Physical Activity**				
General physical activity (cpm)	757.2 ± 257.4	649.7 ± 193.0	711.7 ± 186.5	587.9 ± 116.4
**Biologial Factors**				
Age	9.7 ± 0.6	9.7 ± 0.6	9.9 ± 0.6	9.9 ± 0.6
Body height (cm)	140.3 ± 6.8	139.5 ± 7.4	140.7 ± 7.2	140.3 ± 8.6
Body weight (kg)	35.3 ± 8.2	34.9 ± 8.0	33.8 ± 7.1	33.7 ± 7.2
Body mass index (kg/m^2^)	17.8 ± 3.2	17.8 ± 3.3	17.0 ± 2.5	17.0 ± 2.2
Fat mass (% of whole body)	18.2 ± 10.3	24.6 ± 9.8	15.9 ± 7.3	21.2 ± 8.0
Lean mass (% of whole body)	78.0 ± 10.1	71.8 ± 9.4	80.2 ± 7.1	75.0 ± 7.7
Born during January–June *n* (%)	30 (43)	19 (44)	22 (44)	18 (39)
**Social Factors**				
Child spend more than 14 h/week on sedentary activity, *n* (%)	46 (66)	19 (44)	32 (64)	28 (61)
Having minimum one parent with university degree, *n* (%)	45 (64)	33 (77)	31 (62)	31 (67)
Having minimum one parent that totally agree that exercise is important, *n* (%)	44 (63)	21 (49)	31 (62)	33 (71)
Parental duration of PA (h/week)	2.9 ± 2.2	2.7 ± 2.2	3.1 ± 2.4	2.2 ± 1.2
Have sibling member of a sport association, *n* (%)	44 (63)	25 (58)	22 (44)	29 (63)
**Environmental Factors**				
Living in a house, *n* (%)	66 (94)	39 (91)	20 (40)	18 (40)

**Table 2:** Association between included socio-ecological factors and objectively measured general physical activity unadjusted and adjusted for the other factors included in the model, 2 years after baseline in the POP study.

	Descriptive Statistics	Regression Coefficient (95%CI) Unadjusted	Regression Coefficient (95%CI) Adjusted
**Biological Factors**			
Male sex, *n* (%)	120 (57)	Reference	Reference
Female sex, *n* (%)	89 (43)	− 120.5 (−176.7, −64.3) ***	−66.1 (−122.9, −9.3) *
Age, years ± SD	9.8 ± 0.6	−13.4 (−60.2, 33.3)	−54.8 (−108.2, −1,4) *
Born January–June, *n* (%)	89 (43)	Reference	Reference
Born July–December, *n* (%)	120 (57)	−1.7 (−60.3, 56.9)	13.9 (−43.5, 71.3)
Body height, cm ± SD^¤^	140.2 ± 7.4	19 (−20.0, 58.1)	69.0 (24.6, 113,3) **
Fat mass, % of whole body ± SD	19.6 ± 9.5	−7.5 (−10.4, −4.7) ***	−55.1 (−157.4, 47.2)
Lean mass, % of whole body ± SD	76.6 ± 9.3	7.7 (4.7, 10.6)***	−48.8 (−154.2, 56.5)
**Social Factors**			
Child spend more than 14 h/week on sedentary activity, *n* (%)	125 (60)	Reference	Reference
Child spend less than 14 h/week on sedentary activity, *n* (%)	69 (33)	44.9 (−17.6, 107.5)	39.5 (−16.8, 95.7)
Unknown, *n* (%)	15 (7)	−27.6 (−141.6, 86.4)	30.6 (−75,4, 136.6)
Having minimum one parent with university degree, *n* (%)	140 (67)	Reference	Reference
Having no parent with university degree, *n* (%)	69 (33)	58.1 (−3.0, 119.2)	43.7 (−12.5, 99.8)
No parent totally agree that exercise is important, *n* (%)	65 (31)	Reference	Reference
Minimum one parent that totally agree that exercise is important, *n* (%)	129 (62)	58.3 (−4.5, 121.0)	46.1 (−14.1, 106.3)
Unknown, *n* (%)	15 (7)	−72.0 (−190.2, 46.2)	−79.2 (−189.3, 30.8)
Parental duration of PA (h/week ± SD)	2.7 ± 2.1	14.4 (0.6, 28.1) *	10.3 (−2.4, 23.1)
Have no sibling, *n* (%)	89 (43)	Reference	Reference
Have sibling member of a sport association, *n* (%)	120 (57)	55.0 (−3.1, 113.1)	9.0 (−49.8, 67.8)
**Environmental Factors**			
Have 60 min school PA/week, *n* (%)	96 (46)	Reference	Reference
Have 40 min daily school PA, *n* (%)	113 (54)	63.9 (6.5, 121.4) *	81.8 (15.7, 147.8) *
Living in an apartment, *n* (%)	66 (32)	Reference	Reference
Living in a house, *n* (%)	143 (68)	54.4 (−7.5, 116.2)	−21.6 (−92.2, 49.1)

*p* < 0.05*; *p* < 0.01**; *p* < 0.001***; per ten-unit change.

## Appendix A

**Table A1:** Description of factors included in the model.

Factor included in the Model	Query Formulation in Questionnaire	Comment
Sex	Boy or girl?	
Age	Date of birth	
Relative age	Date of birth	We divided the answers into born during January–June or July–December.
Children’s screen time activity (h/week)	How many hours per day do you spend in front of the TV or computer? (h/day)	Answers were transformed to h/week. We then categorized children above/below median (14 h/week). Missing values were categorized as “unknown”.
Parental educational level	What is your mother/father’s highest educational level? Compulsory school High school University/College	The mother and father answered these questions separately and chose one of the three answer options. We categorized the answers to: Having minimum of one parent with university/college degree None of the parents have university/college degree If only one parent answered and the answer was having a university/college degree, then we could assume a minimum of one parent with university/college/degree. If only one parent answered any of the other three options, we did not include the answer in the analysis.
Parental attitude towards physical activity	In our family, it is important to exercise Agree Partly agree Do not agree	The mother and father answered this question separately and chose one of the three answer options. We categorized the answers into: Both, or one of the parents totally agree, None of the parents totally agree, Unknown
Parental physical activity levels (h/week)	How many hours per week is your mother/father physically active during leisure time with, for example, cycling or other sports?	The mother and father answered this question separately. Thereafter a mean value of mothers and fathers PA (h/week) was calculated. If only one parent answered, we used this answer as the mean value for both parents (*n* = 6).
Siblings	Do you have any siblings? Yes No If you have sibling(s), is your sibling(s) active in any sport association? Yes No	We categorized the answers into: Have sibling active in sport association, Do not have sibling active in sport association. Category 2 included those having a sibling that was not active in a sport association and those not having any siblings.
Daily School PA		Children were categorized to (i) intervention or (ii) control, depending on which school they attended.
Home	How do you live? House Apartment Other, specify	We categorized the answers into Living in house Living in apartment If the children answered (iii) we converted their answer to the most suitable option of house or apartment (*n* = 7).
